# “Triple positive” early breast cancer: an observational multicenter retrospective analysis of outcome

**DOI:** 10.18632/oncotarget.7480

**Published:** 2016-02-18

**Authors:** Patrizia Vici, Laura Pizzuti, Isabella Sperduti, Antonio Frassoldati, Clara Natoli, Teresa Gamucci, Silverio Tomao, Andrea Michelotti, Luca Moscetti, Stefania Gori, Editta Baldini, Francesco Giotta, Alessandra Cassano, Daniele Santini, Diana Giannarelli, Luigi Di Lauro, Domenico Cristiano Corsi, Paolo Marchetti, Valentina Sini, Domenico Sergi, Maddalena Barba, Marcello Maugeri-Saccà, Michelangelo Russillo, Lucia Mentuccia, Loretta D'Onofrio, Laura Iezzi, Angelo Fedele Scinto, Lucia Da Ros, Ilaria Bertolini, Maria Luisa Basile, Valentina Rossi, Ruggero De Maria, Filippo Montemurro

**Affiliations:** ^1^ Division of Medical Oncology 2, “Regina Elena” National Cancer Institute, Rome, Italy; ^2^ Biostatistics Unit, “Regina Elena” National Cancer Institute, Rome, Italy; ^3^ Division of Oncology, S. Anna Hospital, Ferrara, Italy; ^4^ Department of Experimental and Clinical Sciences, University “G. d'Annunzio”, Chieti, Italy; ^5^ Medical Oncology Unit ASL Frosinone, Frosinone, Italy; ^6^ Department of Medico-Surgical Sciences and Biotechnologies, “Sapienza” University of Rome, Oncology Unit, Istituto Chirurgico Ortopedico Traumatologico, Latina, Italy; ^7^ Oncology Unit I, Azienda Ospedaliera Universitaria Pisana, Pisa, Italy; ^8^ Department of Oncology, Division of Medical Oncology, Belcolle Hospital, ASL Viterbo, Viterbo, Italy; ^9^ Department of Oncology, Ospedale Sacro Cuore Don Calabria, Negrar, Verona, Italy; ^10^ Department of Medical Oncology, S. Luca Hospital, Lucca, Italy; ^11^ Division of Medical Oncology, IRCCS, Giovanni Paolo II Hospital, Bari, Italy; ^12^ Medical Oncology, Catholic University of Sacred Heart, Rome, Italy; ^13^ Department of Medical Oncology, University Campus Bio-Medico, Rome, Italy; ^14^ Medical Oncology, Ospedale San Giovanni Calibita Fatebenefratelli, Rome, Italy; ^15^ Oncology Unit, Sant'Andrea Hospital, “Sapienza” University of Rome, Rome, Italy; ^16^ Medical Oncology, S. Spirito Hospital, Rome, Italy; ^17^ Scientific Direction, “Regina Elena” National Cancer Institute, Rome, Italy; ^18^ Department of Molecular Medicine, “Umberto I”, “Sapienza” University of Rome, Roma, Italy; ^19^ Investigative Clinical Oncology, Fondazione del Piemonte per l'Oncologia-Candiolo Cancer Institute (IRCCs), Candiolo, Italy; ^20^ Division of Medical Oncology, Ospedale Civile di Saluzzo, Saluzzo, Italy

**Keywords:** triple positive, adjuvant breast cancer, trastuzumab, chemotherapy, hormonal receptors

## Abstract

We recently found that trastuzumab benefit may be lower in a small subset of early breast cancer (BC) patients (pts) with tumors expressing high levels of both hormonal receptors (HRs), i.e. triple positive (TP). To better investigate the role of HRs in HER2 positive BC, we retrospectively identified 872 TP BC pts treated with adjuvant chemotherapy alone (cohort A-366 pts), or plus trastuzumab (cohort B-506 pts). Relapse-free-survival (RFS) and breast-cancer-specific-survival (BCSS) were evaluated. Trastuzumab improved RFS and BCSS in all the subsets analyzed, but the effect on BCSS in tumors expressing both HRs in >30% of cells (TP30), and even on RFS in tumors with both HRs expressed in >50% of cells (TP50) was not significant. Distinct patterns of relapse were observed in TP50 and no-TP50 tumors, the former showing low and constant risk in the first 5 years, a late increase beyond 5 years and modest trastuzumab effect. Trastuzumab effect tended to disappear in pts whose tumors expressed ER in >50% of cells. Multivariate analysis of RFS confirmed a significant interaction between trastuzumab and ER expression, with benefit confined to pts whose tumors expressed ER in ≤50% of cells.

Our data suggest that the pattern of relapse of TP tumors with high HRs is similar to that of “luminal”, HER2 negative tumors, without clear benefit from adjuvant trastuzumab, which remains the standard treatment even in TP tumors. Confirmatory findings on the extent to which quantitative expression of HRs may impact clinical behavior of HER2 positive BC are warranted.

## INTRODUCTION

Approximately 25% of breast cancers overexpress the human epidermal growth factor receptor 2 (HER-2) and have an aggressive clinical behaviour [1]. About half of HER-2 positive breast cancer also express hormone receptors (HRs), even if HER-2 positive tumors often, though not always, express HRs at lower levels compared with HR positive/HER-2 negative tumors [2]. Overall, the use of trastuzumab combined with chemotherapy has dramatically improved prognosis in all stages of HER-2 positive breast cancer [3–5]. There has been a general consensus in fact, that the HER-2 oncogene, when overexpressed, is the dominant driver of breast cancer biology, regardless of HR status [6], and current guidelines support the use of anti-HER-2 agents combined with chemotherapy in all the disease settings. Even though treatment with HER-2 targeted agents in all stages of HER-2 positive breast cancer has shown benefit independently on HR status, it is now being increasingly clear that the magnitude of such benefit may differ by HR status. In the adjuvant setting recent reports of outcomes by tumor subtype demonstrated that HR positive/HER-2 positive tumors show usually different behavior and response to therapy, as well as distinct timing and pattern of relapse in comparison to HR negative/HER-2 positive tumors [6–10]. Yet, no data on outcome are specifically reported on tumors expressing HER-2 and both HRs, i.e., estrogen receptor (ER) and progesterone receptor (PgR). The subset of “triple positive” (TP: ER/PgR/HER-2 positive) tumors might represent a distinct entity with a particularly favourable prognosis, and the combination of chemotherapy, HER-2 blockade and endocrine treatment, in some instances, might be considered an overtreatment. Thus far, only few studies evaluated the degree of HRs expression in HER-2 positive disease, together with its influence on treatment efficacy and prognosis [11, 12] and, to our knowledge, no study has focused on TP treatment outcomes.

In a recent work we analyzed data from a large historic cohort of HER-2 positive early breast cancer patients treated with chemotherapy alone or with sequential trastuzumab at various Italian cancer centres between 1998 and 2011. Results were in key with a clear benefit of adding trastuzumab to adjuvant chemotherapy in all breast cancer subsets, including TP tumors, with the unique exception of a small cohort of tumors expressing very high levels (≥50%) of both HRs (TP50) [13].

In order to better define and interpret the outcome of adjuvant treatment specifically in this relevant subset, we expanded our initial cohort of TP, early breast cancer patients treated with chemotherapy with or without trastuzumab in the routine clinical practice by adding data collected in nineteen Italian oncologic centres.

## RESULTS

### Patient and tumor characteristics

Overall, we retrospectively identified 872 consecutive, TP positive, early breast cancer patients treated in routine clinical practice in nineteen Italian cancer centers between January 1998 and December 2011.

Main baseline patient and tumor characteristics are reported in Table 1. Overall, the *HER-2* gene was overexpressed (3+) in 716 (82.1%) and amplified in 156 tumors (17.9%). Among the patients enrolled, 366 (41.9%) and 506 (58%) patients received adjuvant chemotherapy without (cohort A) and with trastuzumab (cohort B), respectively. Median age and menopausal status were well balanced between the two cohorts. Conversely, patients in cohort B had more frequently higher stage, grade 3 and high Ki67 tumors, compared with those in cohort A.

**Table 1:** Descriptive characteristics of the study population (N=872)

Characteristic	N (%)		p
	Cohort A (366 pts)	Cohort B (506 pts)	
**Age (years)**			
Median	52	53	0.54
Range	(29-85)	(26-79)	
**Menopausal status**			
Pre	167 (45.6)	220 (43.5)	0.53
Post	199 (54.4)	286 (56.5)	
**Histology**			
Ductal	311 (84.9)	460 (90.9)	0.006
Lobular	28 (7.7)	24 (4.7)	
Other	27 (7.4)	22 (4.4)	
**Tumor size**			
T1	199 (54.4)	335 (66.2)	0.005
T2	155 (42.3)	157 (31)	
T3	8 (2.2)	9 (1.8)	
T4	4 (1.1)	5 (1)	
**Nodal status**			
N0	151 (41.3)	250 (49.4)	0.02
N1	118 (32.2)	162 (32)	
N2	58 (15.8)	61 (12.1)	
N3	39 (10.7)	33 (6.5)	
**Clinical stage**			
I	111 (30.3)	201 (39.7)	<0.0001
II	146 (39.9)	211 (41.7)	
III	109 (29.8)	94 (18.6)	
**Grading**			
G1	15 (4.1)	17 (3.4)	0.001
G2	167 (45.6)	181 (35.8)	
G3	163 (44.5)	292 (57.7)	
Unknown	21 (5.7)	16 (3.2)	
**Ki67**			
≤14%	72 (19.7)	83 (16.4)	<0.0001
>14%	262 (71.6)	407 (80.4)	
Unknown	32 (8.7)	16 (3.2)	
**Multifocality**			
Unifocal	240 (65.6)	369 (72.9)	0.04
Multifocal	88 (24)	103 (20.4)	
Unknown	38 (10.4)	34 (6.7)	
**HER2 status**			
Overexpressed (3+)	313 (85.5)	403 (79.6)	0.02
Amplified	53 (14.5)	103 (20.4)	
**Hormone receptor status**			
TP ER 1-30 tumors	55 (15)	49 (9.8)	
TP ER 31-50 tumors	53 (14.5)	42 (8.3)	
TP ER 51-70 tumors	68 (18.6)	103 (20.3)	0.001
TP ER 71-100 tumors	190 (51.9)	312 (61.6)	
TP PgR 1-30 tumors	134 (36.2)	184 (36.4)	
TP PgR 31-50 tumors	60 (16.0)	83 (16.4)	0.99
TP PgR 51-70	69 (18.7)	95 (18.7)	
tumors TP PgR 71-100 tumors	103 (29.1)	144 (28.5)	
TP50	147 (40.2)	223 (44.1)	
No TP 50	219 (59.8)	283 (55.9)	0.25
TP30	214 (58.5)	314 (62.1)	0.29
No TP30	152 (41.5)	192 (37.9)	

Abbreviations: Pts, patients; TP ER 1-30, triple positive tumors (HER2/ER/PgR-positive) with ER staining in 1-30% tumor cells; TP ER 31-50, TP tumors with ER staining in 31-50% tumor cells; TP ER 51-70, TP tumors with ER staining in 51-70% tumor cells; TP ER 71-100, TP tumors with ER staining in 71-100% tumor cells; TP PgR 1-30, TP tumors with PgR staining in 1-30% tumor cells; TP PgR 31-50, TP tumors with PgR staining in 31-50% tumor cells; TP PgR 51-70, TP tumors with PgR staining in 51-70% tumor cells; TP PgR 71-100, TP tumors with PgR staining in 71-100% tumor cells; TP30, TP tumors with both ER and PgR staining in more than 30% tumor cells; TP50, TP tumors with both ER and PgR staining in more than 50% tumor cells.

### Adjuvant treatment

Data on adjuvant treatments are reported in Table 2. Overall, among 872 enrolled patients, 34.4% patients received anthracycline-based, 8.4% taxane-based, 44.8% anthracycline+taxane-based, and 12.4% anthracycline-taxane-free chemotherapy. Anthracycline-based regimens were more common in cohort A (51.4%) than in cohort B (22.1%). Conversely, more patients received anthracycline+taxanes-based regimens in cohort B (62.5%), compared to cohort A (20.2%). A comparable proportion of patients in both cohorts received 6 or more cycles of adjuvant chemotherapy (71.3% and 78.3% in cohort A and B, respectively). Trastuzumab was given sequentially in 28% of the patients, and concomitantly with chemotherapy in 72% of the patients. The majority of patients (93.2%) received the three-weekly schedule of trastuzumab (8 mg/kg loading dose, followed by three-weekly doses of 6 mg/kg), with a median duration of 52 weeks (range, 6-75). All but 70 patients (8%) started standard adjuvant hormonal therapy upon completion of chemotherapy. Radiotherapy was given when indicated, according to the Institutional guidelines.

**Table 2:** Treatment administered to the study population (N=872)

Characteristics	N (%)		p
	Cohort A (366 pts)	Cohort B (506 pts)	
**Surgery**			
Conservative	214 (58.5)	341 (67.4)	0.007
Radical	152 (41.5)	165 (32.6)	
**Chemotherapy regimens**			
Anthracyclines-based	188 (51.4)	112 (22.1)	<0.0001
Taxanes-based	13 (3.6)	61 (12.1)	
Anthracyclines and Taxanes-based	74 (20.2)	316 (62.5)	
No anthracyclines and No taxanes–based	91 (24.9)	17 (3.4)	
**Cycles of chemotherapy**			
<6	64 (17.5)	78 (15.4)	0.24
≥6	261 (71.3)	396 (78.3)	
Unknown	41 (11.2)	32 (6.3)	
**Radiotherapy**			
Yes	235 (64.2)	373 (73.7)	0.003
Not	131 (35.8)	133 (26.3)	
**Hormonal therapy**			
Yes	332 (90.7)	470 (92.9)	0.24
Not	34 (9.3)	36 (7.1)	

Abbreviations: Pts, patients

### Survival analysis

**Figure 1:**
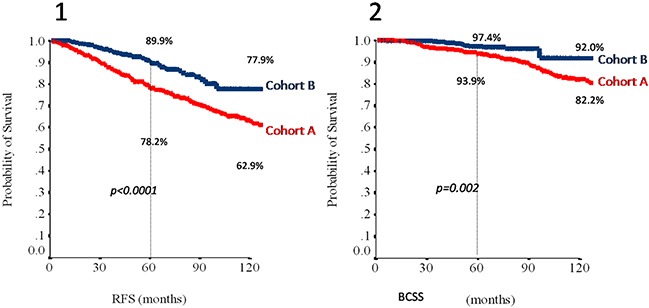
Relapse-free survival (1) and breast cancer specific survival (2) in the overall study population (N=872) according to cohort Abbreviations: RFS, relapse free survival; BCSS, breast cancer specific survival. Cohort A: red line; Cohort B: blue line.

The median follow up for the whole patient series was 78 months (95% CI, 74 to 82), being 123 months (95% CI, 118 to 128) in cohort A and 62 months (95% CI, 59 to 65) in cohort B. Overall, we observed 194 recurrences, 139 in cohort A and 55 in cohort B. Kaplan-Meier estimates of RFS and BCSS according to cohort of treatment in the overall population are shown in Figure 1.

**Figure 2:**
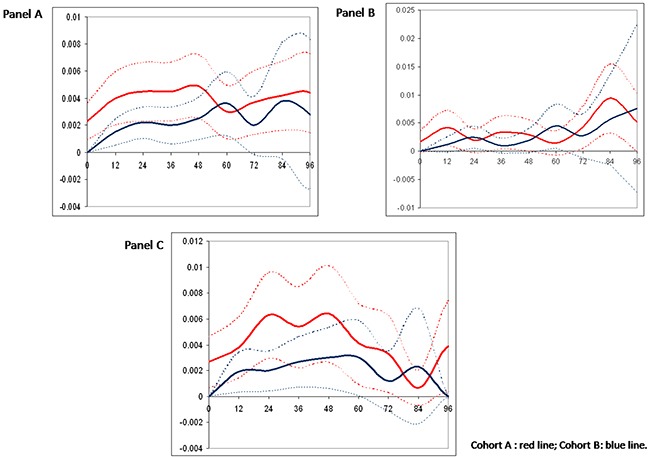
Hazard rate of recurrence for the overall study population (panel A) and in patients with ER and PgR staining in more than 50% of tumour cells (TP50, panel B) and other patients (noTP50, panel C) Cohort A: red line; Cohort B: blue line.

**Figure 3:**
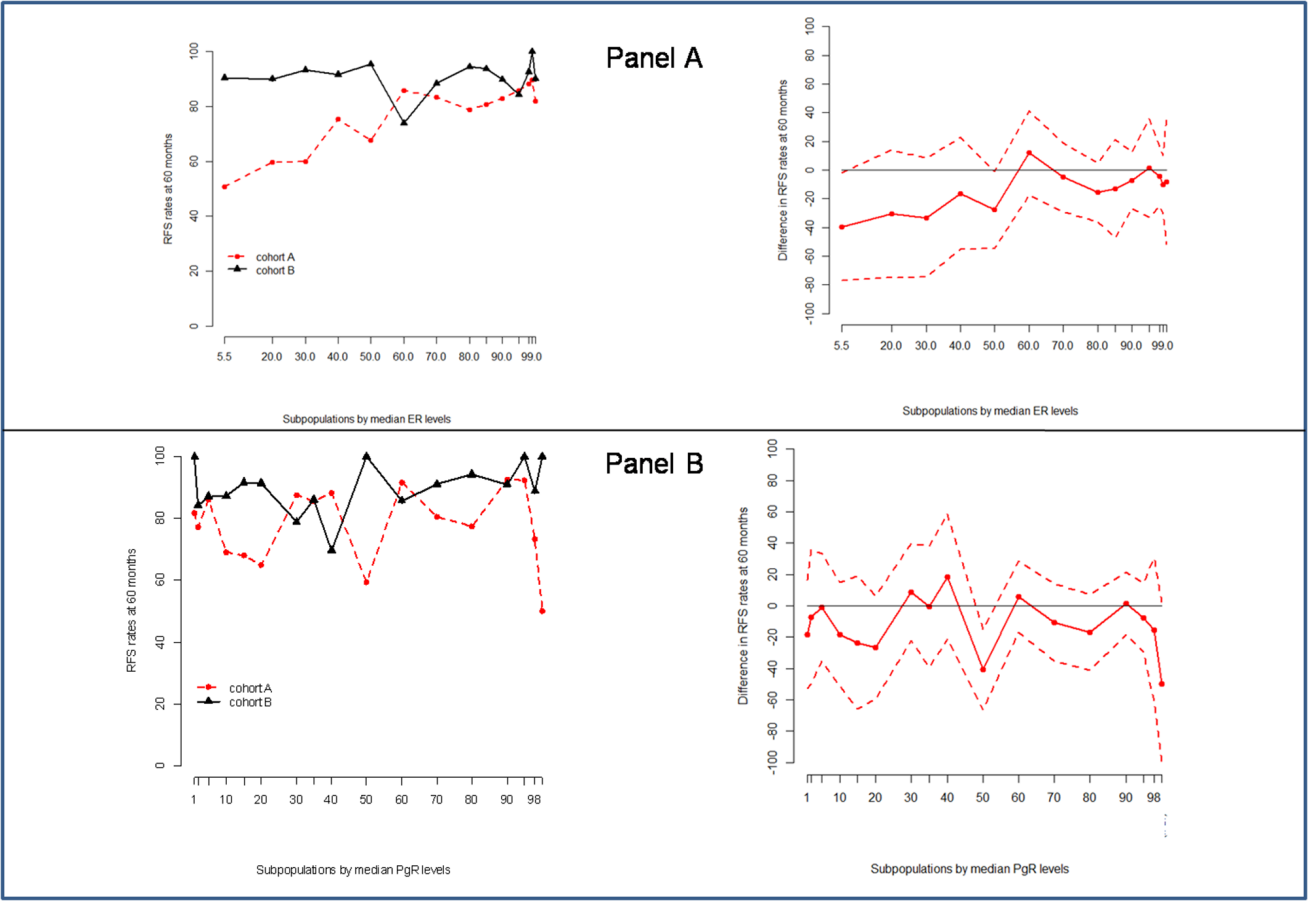
Stepp analysis of the effect on ER (Panel A) and PgR (Panel B) expression on the hazard of relapse in the two cohorts Abbreviations: ER, estrogen receptor; PgR, progesterone receptor; RFS, relapse free survival.

To study the effect of different cutoffs of HRs expression in TP tumors we defined a TP30 (both ER and PgR expressed in >30% of tumor cells) and a TP50 (both ER and PgR expressed in >50% of tumor cells) populations. Estimates of 3- and 5-year RFS and BCSS in the overall, the TP30 and the TP50 populations are summarized in Table 3. Adjuvant trastuzumab (cohort B) was associated with a RFS and BCSS benefit in the overall study population. In addition, no differences were observed in outcomes depending on treatment schedule (weekly or three-weekly, concomitant or sequential with chemotherapy), and use of regimens including anthracyclines and taxanes (data available upon request). However, the BCSS advantage in the TP30 populations and both the RFS and BCSS advantages in the TP50 populations failed to reach statistical significance (Table 3). To further explore the potential effect of having both ER and PgR expressed in more than 50% of tumor cells, we compared the annualized hazards of RFS for cohort A and B patients in the overall, TP50, and no-TP50 (i.e. remainder patients, whose tumor do not have simultaneous expression of ER and PgR in >50% of tumor cells) populations (Figure 2, panels a, b and c, respectively). In the no-TP50 population, relapse events tended to occur during the initial 5 years at a constant rate and the effect of trastuzumab in reducing relapse events appeared constant over this time-interval (Figure 2, panel c). Conversely, in TP50 patients (Figure 2, panel b) a clear effect of trastuzumab could not be observed and in both cohorts the hazards of relapse tended to increase slightly beyond 5 years of follow-up. We performed a STEPP analysis to relate increasing percentages of cells staining positively for ER (Figure 3, panel a) and for PgR (Figure 3, panel b) with the risk of relapsing at 5 years according to cohort. For ER, we observed a clear reduction in such risk beyond 50% of tumor cells staining positively, whereas no such effect was seen for increasing PgR expression. At multivariate analysis factors related to increased risk of relapse at 5 years were presence of multifocality, high tumor grade, advanced stage at diagnosis, no adjuvant endocrine therapy, no adjuvant trastuzumab and ER≤50%. Since we found a significant 2×2 interaction of ER expression with the effect of trastuzumab (p = 0.01), in the final model we report the effect of trastuzumab across the two strata of the ER50 variable, together with the other variables independently associated with RFS (Table 4). In the ER>50 stratum, trastuzumab showed no significant effect on 5-year RFS.

**Table 3:** Summary of 3 and 5-year relapse-free survival (RFS) and breast cancer specific survival (BCSS) comparisons between Cohort A and Cohort B according to study population

Population	Number	3-year RFS	5-year RFS	HR	P	3-year BCSS	5-year BCSS	HR	P
**Overall**									
**Cohort A**	366	87.0	78.2	0.48	<0.0001	96.3	93.9	0.39	0.001
**Cohort B**	506	95.6	89.9	(0.35-0.67)		99.3	97.4	(0.21-0.73)	
**TP30**									
**Cohort A**	214	89.4	81.4	0.60	0.02	97.1	96.6	0.63	0.26
**Cohort B**	314	95.6	90.7	(0.39-0.93)		99.3	97.5	(0.28-1.43)	
**TP50**									
**Cohort A**	147	90.3	83.7	0.64	0.09	95.7	95.7	0.50	0.16
**Cohort B**	223	95.7	90.6	(0.38-1.07)		100	97.4	(0.19-1.35)	

Abbreviations: Pts, patients; TP 30, triple positive tumors (HER2/ER/PgR-positive) with ER and PgR staining > 30% tumor cells; TP 50, triple positive tumors (HER2/ER/PgR-positive) with ER and PgR staining > 50% tumor cells; HR, hazard ratio.

**Table 4:** Multivariate logistic regression model of factors impacting survival outcomes in the study population

Factors	5 year RFS	
	OR (95%CI)	P-value
Multifocality		
*Yes vs No*	1.96(1.17-3.28)	0.01
Grading		
3 vs 1 - 2	2.26(1.36-3.75)	0.002
Stage		
*III vs I-II*	3.59(2.20-5.85)	<0.0001
Hormonal therapy		
*No vs Yes*	2.88(1. 53-6.14)	0.006
^a^Trastuzumab therapy in ER≤50%		
*No vs Yes*	4.33 (1.78-10.55)	0.001
^a^Trastuzumab therapy in ER50		
*No vs Yes*	1.16 (0.70-1.93)	0.57

Abbreviations: CI, confidence interval; OR, Odds ratio; RFS, Relapse-Free Survival; ER50, TP tumors with ER staining in more than 50% tumor cells

a ER*Trastuzumab therapy, p_INTERACTION_ =0.01

## DISCUSSION

In this retrospective analysis, we sought to expand our previous observation that in patients with operable HER2-positive breast cancer, high co-expression of both the ER and PgR (“triple-positivity”-TP) could mitigate the effect of trastuzumab administered with adjuvant chemotherapy [13]. In fact, in the RETROHER study, we found little benefit of adjuvant trastuzumab in 163 women with HER2-positive operable breast cancer whose tumors were positive for both the ER and PgR in ≥50% of tumor cells. We thought that this initial observation deserved further investigation. To better study the influence of degree and pattern of HRs expression on clinical behavior and trastuzumab efficacy, we recruited 872 patients with TP breast cancer selected on the basis of a HER2-positive status and simultaneous expression of both ER and PgR in at least 1% of the tumor cells. Our first finding was that hazard ratios for RFS and BCSS were consistently in favor of trastuzumab in the overall, TP30 and TP50 populations, although differences failed to achieve statistical significance for BCSS in the TP30, and for RFS and BCSS in the TP50 populations. Five-year absolute, trastuzumab-related RFS gains were 9.5% and 6.9% for TP30 and TP50 patients respectively, with apparently no effect on BCSS (0.9% and 1.7% for TP30 and TP50 patients, respectively). Corresponding figures in the overall population were 11.7% and 3.4% for RFS and BCSS, respectively (Table 3). A second finding of our study was that the annualized hazard of relapse in TP50 patients differs profoundly from that of patients whose tumors do not have simultaneous expression of ER and PgR in >50% of tumor cells (No-TP50) (Figure 2). Furthermore, in this latter group of patients, a favorable effect of trastuzumab was seen early and persisted for 4-6 years from surgery. Conversely, for TP50 patients the annualized hazard of disease relapse tended to remain low and constant for about 6 years from surgery and to increase thereafter, with no clear pattern regarding the effect of trastuzumab. The third finding of our study was that expression of the ER in >50% of tumor cells was associated with a potential reduction in the effect of trastuzumab on RFS, and a statistically significant interaction could be demonstrated at the multivariate analysis (Table 4). Taken together, these results suggest that trastuzumab added to chemotherapy must remain a standard of care in patients with HER2 positive tumors, regardless hormone receptor status. However, quantitative expression of HRs may influence the clinical behavior of HER2-positive breast cancer, with potentially relevant implications in the estimation of risk and timing of relapse and, finally, on treatment choices.

In the interpretation of these results we must consider obvious biases that are related to the retrospective methodology that we used. Cohort A and B were sequential and not parallel, resulting in median follow-up in cohort A being twice as long as that of patients in cohort B. Consequently, more disease relapses and breast cancer deaths may have occurred in cohort A just because of longer observation time. Although HER2-positivity confers early metastatic potential, long term follow-up of the adjuvant HERA trial shows events beyond 5 years in patients receiving trastuzumab [14]. Therefore, we could not exclude that 60 months of median follow-up of cohort B of our study is insufficient to capture all the events.

Furthermore, because of the non-randomized design, some imbalances were seen in cohort A and B with respect to histopathological features. Although more frequently high-stage, high-grade and highly proliferating, tumors in cohort B were also more frequently high in HRs expression than patients in cohort A (Table 1). This is another factor that may have biased the comparison of outcomes between the two cohorts.

Finally, and perhaps most importantly, the ER/PgR expression level is just a surrogate for molecular subtyping, and quantitative analysis of ER and PgR status was not centralized, but extracted from the medical charts of the patients at each participating Institution. As a consequence, inter-laboratory variability and bias cannot be excluded and should be taken into account in interpreting our results [15].

Several adjunctive variables, not investigated in the present study, such as the level of stromal Lymphocyte Tumor Infiltration (sTIL) and PIK3CA mutations, EGFR or PTEN expression, along with distinct intrinsic molecularly-defined subtypes, may have influenced outcomes [16–21]. Despite the numerous potential biases, we believe in the biological and clinical plausibility of our results and in their potential implications for further research in this area.

In HER2-negative breast cancer, a number of studies have confirmed that late disease-related events are common in HRs positive patients [22]. This translates into a time-dependent hazard ratio for HRs positive *vs* HRs negative tumors [23, 24]. This same phenomenon was recently observed in women enrolled in the adjuvant Tykerb Evaluation after Chemotherapy (TEACH) clinical trial [25], where patients with HER2 positive operable breast cancer not receiving adjuvant trastuzumab were randomized to adjuvant lapatinib or observation at a median time from diagnosis of 70 months. A recent report focusing on patients assigned to the placebo arm revealed that HR status (positive *vs* negative) had a time-varying effect on the hazard of disease relapse [26]. HRs positive tumors showed a slowly decreasing hazard compared with HRs negative tumors, which showed a higher and more rapidly declining hazard of disease-relapse during follow-up. At approximately 6 years, the two annualized hazard curves tended to cross. This observation confirms that HR status (positive *vs* negative) may influence clinical behavior also in HER2-positive breast cancer.

Since high expression of ER and PgR is regarded as a proxy for an “endocrine-responsive” behavior we decided to focus our analysis on TP tumors [27]. In line with the previously mentioned results, we could show that, within HER2-positive/HRs positive breast cancers, the expression of either or both ER and PgR in >50% of tumor cells identifies entities that behave like “endocrine-responsive” tumors. Additionally, STEPP analysis could show a sharp reduction in the effect of trastuzumab in tumors expressing the ER in more than 50% of the cells.

An intriguing retrospective evaluation of eight predictive genes associated with HER2 or ER in the NSABP-B31 trial showed that high-levels of ESR1 mRNA expression may be associated to low trastuzumab benefit [28]. We may hypothesize that the high ER expression level in a subset of patients in the present study might be representative of the subgroup with higher ESR1 mRNA expression.

Conversely to widespread belief in the early 2000s, it is now widely recognized that HER2 positive tumors are not homogeneous and that HRs co-expression represents a robust biomarker of both the clinical behavior and the effect of anti-HER2 treatments [29]. This latter factor emerged clearly in the neo-adjuvant setting, when results of the NEO-ALTTO and NEO-SPHERE trials, evaluating neoadjuvant taxanes with anti HER2 treatments in HER2-positive patients, showed a clear reduction in pCR when the tumor co-expressed HRs, mirrored by very high pCR in HER2-positive/HRs negative tumors [30, 31]. Further observations from retrospective studies in the metastatic and neoadjuvant setting also suggested a potential relationship between degree of HRs expression and response to trastuzumab treatment, with reduced activity in patients with high-expression [11, 12]. Additionally, while predicting shorter disease-free survival in HER2-positive/HRs negative tumors, failing to achieve a pCR following neoadjuvant treatment does not preclude favorable long-term outcomes in HER2-positive/HRs positive tumors [32]. This effect on outcome is clearly due to the fact that, despite being acknowledged as a predictor of resistance to endocrine therapy, in the context of HER2 inhibition, HER2-positive/HRs positive tumors can partially recover endocrine-responsiveness [33–35]. Indeed, a retrospective study confirmed long progression-free survival in women assigned to trastuzumab and maintenance endocrine therapy after stable disease or tumor response to first-line chemotherapy and trastuzumab [11]. The biology behind HER2-positive breast cancer provides explanations for this clinical diversity, calling into cross-talk between the HER2 and HRs pathways [29, 32, 36]. Traditionally, this cross-talk has been considered unidirectional, where aberrant HER2 function could provide escape to endocrine therapy. Recent data suggest bidirectional and dynamic cross-talk, where also the HRs pathway could provide escape to HER2-inhibition. A neoadjuvant study of HER2 targeting agents and endocrine therapy showed an increase in pCR when both treatments are administered concomitantly, thus confirming preclinical models [37]. Biological diversity has been recently further clarified in the most comprehensive molecular characterization of breast cancer, which clearly shows that two distinct HER2 positive breast cancer exist [38]. One is the HER2-enriched mRNA subtype, which is characterized by increased expression of tyrosine-kinase receptor like FGFR4, EGFR, and of genes mapping on the HER2 amplicon. The other is the luminal mRNA subtype positive, with increased expression of luminal genes like BCL2, GATA3 and ESR1. One major phenotypical difference between these two subgroups is the expression of hormone receptors, which is rare in the former, and frequent in the latter subtype. Unfortunately, immunohistochemical surrogates of these two distinct biological entities are barely obtainable in the clinic, being the mere positivity for either the ER or PgR a definition that could encompass both subtypes. Therefore, additional markers of the “luminal/HER2-positive” phenotype are awaited to better exploit biological diversity in the clinic. A recent report from Neoadjuvant Breast Symphony trial compared a multigene classifier (Blue-Print 80-gene assay) to conventional IHC/FISH to predict pCR to chemotherapy plus HER2 block. The Blue-Print (BP) assay re-classified more than 1 over 5 tumors, and the group with the biggest change was the TP group, where the BP-defined “luminal” subgroup showed the lower pCR to trastuzumab (11%), compared to that of the BP-defined HER2 (45%); the pCR increased with double-block including pertuzumab [39].

Overall, we provide evidence that the clinical behavior of TP breast cancer could depend upon quantitative expression of HRs, with highly co-expressing tumors behaving in a luminal fashion, i.e., low but constant hazard of relapse in the initial 5 years after surgery, and possibly, slow increase thereafter resulting in late recurrences. Furthermore, increasing expression of ER seems to be associated with reduced trastuzumab effect. We believe, however, that future studies should try to address this issue with appropriate methodology because of the potential implications on treatment tailoring according to the biological diversity of HER2-positive breast cancer.

## MATERIALS AND METHODS

Our cohort included patients with early TP tumors routinely treated in the adjuvant setting in nineteen Italian oncologic centres over a period of thirteen years (from 1998 to 2011). Written informed consent was obtained from all patients. Information on demographics, clinical, histopathological and molecular features, adjuvant therapies and outcomes were retrieved from the patients' medical records. Anonymized data were entered into an ad hoc database. Two cohorts were analyzed: patients who received adjuvant chemotherapy without trastuzumab, mostly until 2005 (cohort A), and patients who received adjuvant chemotherapy followed by or combined with trastuzumab (cohort B) since 2006, when adjuvant trastuzumab was approved in Italy. Endocrine treatment and radiotherapy were sequentially given whenever indicated according to standard guidelines.

Pathology assessment was performed on surgical specimens by the pathologists at the participating centers as per National standards. ER and PgR status were determined at each center by immunohistochemistry according to local standards. Positivity was considered at a cutoff of ≥1 % of tumor cells stained. Ki-67 was tested using the mAb MIB1 (Dako) and regarded high if >14 % of the cell nuclei were immunostained. HER2 overexpression was tested using the polyclonal antibody A0485 (Dako), and was considered positive if grade 3+ staining intensity by immunohistochemistry, or grade 2+ with gene amplification by fluorescence, silver or chromogenic in situ hybridization was detected. Definitions and cutoffs for HER2 positivity were the same as those used in the pivotal trastuzumab adjuvant trials. If missing, the molecular features were centrally evaluated in formalin fixed, paraffin-embedded tissue sections, whenever available. This study was approved by the institutional ethical committee of the coordinating center (Regina Elena National Cancer Institute of Rome) and satellite centers and was conducted in compliance with Helsinki Declaration.

### Statistical analysis

Associations between categorical variables were tested by the Pearson Chi Square test or the Fisher Exact test, when appropriate. Relapse-free survival (RFS, time from surgery to any invasive or non-invasive breast cancer recurrence, either local, regional, contralateral or distant) and breast cancer specific survival (BCSS, time from surgery to death in patients who had developed metastatic disease) where chosen as outcome variables of interest. For both RFS and BCSS, patients dying without prior evidence of metastatic disease were censored at the date of their last follow-up visit. Median follow-up was calculated with the reverse method [40]. Kaplan-Meier curves of RFS and BCSS were compared by the log-rank test, with statistical significance set at p≤0.05. The impact of independent variables of interest on 5-years RFS was studied by univariate and multivariate logistic regression model. Odds Ratios are reported together with their 95% confidence intervals (95% C.I.). For the multivariate analyses we adopted a stepwise regression (forward selection) method by selecting significant variables upon univariate analysis. Enter limit and remove limit were p = 0.10 and p = 0.15, respectively. The annual hazard function, giving the risk of disease at a specified time conditional on remaining disease-free up until that point in time, was estimated by cohorts and by HR status using splines, with separate splines fit for the cohort and by HR status. The subpopulation treatment effect pattern plot (STEPP) analysis was performed to evaluate treatment-effect heterogeneity when HRs expression is measured on a continuous scale. STEPP graphically explores the patterns of treatment effect across overlapping intervals of the HRs values [41]. The SPSS software (SPSS version 21.0, SPSS Inc., Chicago, Illinois, USA) and R-Software (version 3.2.1) were used for statistical evaluations.
